# Tris(tetra­ethyl­ammonium) hydrogen bis­[2-(sulfatosulfan­yl)benzoate]

**DOI:** 10.1107/S1600536809041415

**Published:** 2009-10-17

**Authors:** Yun-Xia Yang, Qi Li, Seik Weng Ng

**Affiliations:** a College of Chemistry, Beijing Normal University, Beijing 100875, People’s Republic of China; b Department of Chemistry, University of Malaya, 50603 Kuala Lumpur, Malaysia

## Abstract

The reaction between tetra­ethyl­ammonium hydroxide and 2,2′-dithio­benzoic acid yields the title compound, 3C_8_H_20_N·H(C_6_H_4_O_5_S_2_)_2_^3−^, the trianion of which comprises two 2-(sulfato­sulfan­yl)benzoate dianions linked across a center of inversion by an acid H atom. One of the cations is disordered about another center of inversion.

## Related literature

For the crystal structures of other aryl­thio­sulfates, see: Boese *et al.* (1999 ▶); Chen *et al.* (2004 ▶).
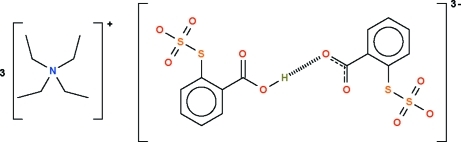


## Experimental

### 

#### Crystal data

3C_8_H_20_N^+^·C_6_H_5_O_5_S_2_^2−^·C_6_H_4_O_5_S_2_^−^
*M* *_r_* = 856.20
Triclinic, 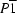

*a* = 7.9774 (1) Å
*b* = 9.2439 (1) Å
*c* = 17.0074 (3) Å
α = 90.649 (1)°
β = 93.845 (1)°
γ = 114.678 (1)°
*V* = 1135.98 (3) Å^3^
*Z* = 1
Mo *K*α radiation
μ = 0.26 mm^−1^
*T* = 293 K
0.50 × 0.20 × 0.20 mm

#### Data collection

Bruker APEXII diffractometer
Absorption correction: multi-scan (*SADABS*; Sheldrick, 1996 ▶) *T*_min_ = 0.880, *T*_max_ = 0.949
10538 measured reflections
5176 independent reflections
4013 reflections with *I* > 2σ(*I*)
*R*_int_ = 0.016

#### Refinement

*R*[*F* ^2^ > 2σ(*F* ^2^)] = 0.058
*wR*(*F* ^2^) = 0.181
*S* = 1.03
5176 reflections
315 parameters
62 restraints
H-atom parameters constrained
Δρ_max_ = 0.96 e Å^−3^
Δρ_min_ = −0.28 e Å^−3^

### 

Data collection: *APEX2* (Bruker, 2007 ▶); cell refinement: *SAINT* (Bruker, 2007 ▶); data reduction: *SAINT*; program(s) used to solve structure: *SHELXS97* (Sheldrick, 2008 ▶); program(s) used to refine structure: *SHELXL97* (Sheldrick, 2008 ▶); molecular graphics: *X-SEED* (Barbour, 2001 ▶); software used to prepare material for publication: *publCIF* (Westrip, 2009 ▶).

## Supplementary Material

Crystal structure: contains datablocks global, I. DOI: 10.1107/S1600536809041415/xu2617sup1.cif

Structure factors: contains datablocks I. DOI: 10.1107/S1600536809041415/xu2617Isup2.hkl

Additional supplementary materials:  crystallographic information; 3D view; checkCIF report

## Figures and Tables

**Fig. 1.**
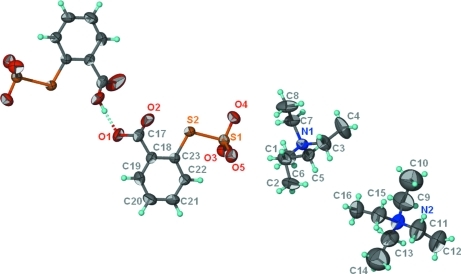
Thermal ellipsoid plot (Barbour, 2001) of 3[(C2H5)4N]+ [H(C6H4O5S2)2]3- at the 50% probability level; hydrogen atoms are drawn as spheres of arbitrary radius. The dashed line denotes a hydrogen bond.

## supplementary crystallographic information

### Comment

The title salt (Fig. 1, Scheme I) was isolated as the product of an attempted hydrolysis of 2,2'-dithiobenzoic acid with tetraethylammonium hydroxide in which an *S*–C_aryl_ bond was cleaved and the free sulfuryl end then oxidized to a sulfonate group.

### Experimental

2,2'-Dithiobenzoic acid (0.25 mmol, 0.08 g) was dissolved in a water-ethanol (1:2 *v*/*v*) mixture. A 25% solution of tetraethylammonium hydroxide was added to neutralize the acid and give a yellow coloration to the solution. Yellow blocks separated after several weeks.

### Refinement

One of the two cations is disordered about a center-of-inversion. The cation was allowed to refine off the special position, and with distance restraints of N–C = C–C = 1.50±0.01 Å and N···C = 2.35±0.01 Å. Their anisotropic temperature factors were restrained to be nearly isotropic.

The two carboxyl oxygen atoms of the anion is disordered over two positions; the occupancy disorder refined to nearly 1:1 and as such, the occupancy of the four oxygen atoms was set as 0.5. One 'acid' hydrogen atom was arbitrarily placed on one of the two carboxyl –CO_2_ components.

Carbon-bound H-atoms were placed in calculated positions (C—H 0.93 to 0.97 Å) and were included in the refinement in the riding model approximation, with *U*(H) set to 1.2*U*(C). The acid H-atom was similar treated.

### Crystal data

**Table:**

3C_8_H_20_N^+^·C_6_H_5_O_5_S_2_^2^^−^·C_6_H_4_O_5_S_2_^−^	*Z* = 1
*M**_r_* = 856.20	*F*(000) = 462
Triclinic, *P*1	*D*_x_ = 1.252 Mg m^−^^3^
Hall symbol: -P 1	Mo *K*α radiation, λ = 0.71073 Å
*a* = 7.9774 (1) Å	Cell parameters from 4127 reflections
*b* = 9.2439 (1) Å	θ = 2.2–28.2°
*c* = 17.0074 (3) Å	µ = 0.26 mm^−^^1^
α = 90.649 (1)°	*T* = 293 K
β = 93.845 (1)°	Block, yellow
γ = 114.678 (1)°	0.50 × 0.20 × 0.20 mm
*V* = 1135.98 (3) Å^3^	

### Data collection

**Table:**

Bruker APEXII diffractometer	5176 independent reflections
Radiation source: fine-focus sealed tube	4013 reflections with *I* > 2σ(*I*)
graphite	*R*_int_ = 0.016
φ and ω scans	θ_max_ = 27.5°, θ_min_ = 2.4°
Absorption correction: multi-scan (*SADABS*; Sheldrick, 1996)	*h* = −8→10
*T*_min_ = 0.880, *T*_max_ = 0.949	*k* = −11→12
10538 measured reflections	*l* = −22→22

### Refinement

**Table:**

Refinement on *F*^2^	Primary atom site location: structure-invariant direct methods
Least-squares matrix: full	Secondary atom site location: difference Fourier map
*R*[*F*^2^ > 2σ(*F*^2^)] = 0.058	Hydrogen site location: inferred from neighbouring sites
*wR*(*F*^2^) = 0.181	H-atom parameters constrained
*S* = 1.03	*w* = 1/[σ^2^(*F*_o_^2^) + (0.1007*P*)^2^ + 0.4839*P*] where *P* = (*F*_o_^2^ + 2*F*_c_^2^)/3
5176 reflections	(Δ/σ)_max_ = 0.001
315 parameters	Δρ_max_ = 0.96 e Å^−^^3^
62 restraints	Δρ_min_ = −0.28 e Å^−^^3^

### Fractional atomic coordinates and isotropic or equivalent isotropic displacement parameters (Å^2^)

**Table:**

	*x*	*y*	*z*	*U*_iso_*/*U*_eq_	Occ. (<1)
S1	0.37903 (8)	0.68955 (7)	0.65535 (4)	0.04844 (19)	
S2	0.44246 (9)	0.81719 (10)	0.76485 (4)	0.0641 (2)	
O1	0.6914 (7)	1.0046 (7)	0.9874 (2)	0.0780 (13)	0.50
O2	0.5551 (8)	1.0673 (8)	0.8853 (3)	0.0895 (16)	0.50
O1'	0.4910 (7)	0.9213 (7)	0.9244 (3)	0.0896 (15)	0.50
H1'	0.4299	0.9501	0.9524	0.134*	0.50
O2'	0.7394 (9)	1.1516 (7)	0.9423 (4)	0.123 (2)	0.50
O3	0.3824 (3)	0.5381 (2)	0.66859 (14)	0.0691 (5)	
O4	0.1964 (3)	0.6831 (3)	0.63761 (13)	0.0711 (6)	
O5	0.5132 (3)	0.7840 (2)	0.60344 (11)	0.0626 (5)	
N1	0.2613 (3)	0.7183 (2)	0.37055 (12)	0.0483 (5)	
C1	0.4500 (4)	0.8384 (3)	0.4057 (2)	0.0648 (7)	
H1A	0.4515	0.9438	0.4049	0.078*	
H1B	0.4650	0.8149	0.4605	0.078*	
C2	0.6126 (4)	0.8418 (4)	0.3648 (3)	0.0862 (11)	
H2A	0.7242	0.9250	0.3886	0.129*	
H2B	0.5977	0.8616	0.3100	0.129*	
H2C	0.6197	0.7411	0.3695	0.129*	
C3	0.2295 (5)	0.7693 (4)	0.2883 (2)	0.0802 (10)	
H3A	0.2444	0.8788	0.2921	0.096*	
H3B	0.3246	0.7666	0.2564	0.096*	
C4	0.0438 (6)	0.6697 (6)	0.2463 (3)	0.1221 (19)	
H4A	0.0316	0.7160	0.1972	0.183*	
H4B	−0.0520	0.6658	0.2786	0.183*	
H4C	0.0331	0.5637	0.2362	0.183*	
C5	0.2520 (4)	0.5528 (3)	0.36561 (16)	0.0577 (6)	
H5A	0.1313	0.4817	0.3414	0.069*	
H5B	0.3437	0.5523	0.3311	0.069*	
C6	0.2829 (5)	0.4878 (4)	0.44214 (18)	0.0700 (8)	
H6A	0.2831	0.3853	0.4328	0.105*	
H6B	0.1856	0.4772	0.4751	0.105*	
H6C	0.3998	0.5590	0.4679	0.105*	
C7	0.1166 (4)	0.7199 (4)	0.4235 (2)	0.0670 (7)	
H7A	−0.0016	0.6353	0.4048	0.080*	
H7B	0.1479	0.6951	0.4762	0.080*	
C8	0.0934 (6)	0.8724 (5)	0.4290 (3)	0.1058 (15)	
H8A	0.0017	0.8618	0.4650	0.159*	
H8B	0.0546	0.8954	0.3778	0.159*	
H8C	0.2092	0.9577	0.4476	0.159*	
C17	0.6587 (4)	1.0166 (3)	0.91364 (15)	0.0567 (6)	
C18	0.7597 (3)	0.9504 (3)	0.86229 (13)	0.0460 (5)	
C19	0.9423 (4)	0.9817 (4)	0.88510 (17)	0.0644 (7)	
H19	0.9974	1.0416	0.9316	0.077*	
C20	1.0444 (4)	0.9273 (4)	0.8414 (2)	0.0723 (8)	
H20	1.1664	0.9496	0.8582	0.087*	
C21	0.9646 (4)	0.8399 (4)	0.77289 (19)	0.0692 (8)	
H21	1.0338	0.8053	0.7420	0.083*	
C22	0.7832 (4)	0.8030 (4)	0.74946 (17)	0.0616 (7)	
H22	0.7296	0.7400	0.7037	0.074*	
C23	0.6766 (3)	0.8578 (3)	0.79291 (14)	0.0459 (5)	
N2	0.5156 (17)	0.4964 (13)	0.0090 (6)	0.068 (2)	0.50
C9	0.3158 (14)	0.3911 (13)	0.0188 (9)	0.137 (4)	0.50
H9A	0.2809	0.2935	−0.0129	0.164*	0.50
H9B	0.3039	0.3626	0.0735	0.164*	0.50
C10	0.182 (3)	0.462 (3)	−0.0034 (13)	0.206 (8)	0.50
H10A	0.1401	0.4900	0.0434	0.309*	0.50
H10B	0.0784	0.3853	−0.0353	0.309*	0.50
H10C	0.2425	0.5553	−0.0327	0.309*	0.50
C11	0.552 (2)	0.5441 (12)	−0.0741 (5)	0.123 (3)	0.50
H11A	0.6746	0.6299	−0.0735	0.148*	0.50
H11B	0.4646	0.5871	−0.0924	0.148*	0.50
C12	0.539 (2)	0.4172 (17)	−0.1343 (7)	0.143 (5)	0.50
H12A	0.6573	0.4138	−0.1353	0.214*	0.50
H12B	0.5023	0.4420	−0.1855	0.214*	0.50
H12C	0.4488	0.3155	−0.1202	0.214*	0.50
C13	0.6031 (14)	0.3908 (11)	0.0405 (6)	0.107 (3)	0.50
H13A	0.5483	0.3471	0.0889	0.129*	0.50
H13B	0.5723	0.3023	0.0027	0.129*	0.50
C14	0.8051 (18)	0.467 (2)	0.0567 (13)	0.198 (8)	0.50
H14A	0.8619	0.5093	0.0091	0.298*	0.50
H14B	0.8477	0.3905	0.0760	0.298*	0.50
H14C	0.8378	0.5527	0.0957	0.298*	0.50
C15	0.5711 (13)	0.6447 (9)	0.0597 (4)	0.092 (2)	0.50
H15A	0.6954	0.7176	0.0489	0.110*	0.50
H15B	0.4894	0.6950	0.0444	0.110*	0.50
C16	0.5675 (16)	0.6226 (15)	0.1450 (5)	0.101 (3)	0.50
H16A	0.4429	0.5586	0.1573	0.152*	0.50
H16B	0.6121	0.7246	0.1725	0.152*	0.50
H16C	0.6450	0.5702	0.1609	0.152*	0.50

### Atomic displacement parameters (Å^2^)

**Table:**

	*U*^11^	*U*^22^	*U*^33^	*U*^12^	*U*^13^	*U*^23^
S1	0.0447 (3)	0.0460 (3)	0.0508 (3)	0.0161 (2)	−0.0003 (2)	−0.0046 (2)
S2	0.0499 (4)	0.0960 (6)	0.0557 (4)	0.0415 (4)	−0.0034 (3)	−0.0234 (3)
O1	0.097 (3)	0.122 (4)	0.0400 (19)	0.072 (3)	0.0035 (19)	−0.007 (2)
O2	0.121 (4)	0.140 (5)	0.058 (2)	0.105 (4)	0.004 (3)	−0.009 (3)
O1'	0.080 (3)	0.124 (4)	0.071 (3)	0.050 (3)	0.014 (2)	−0.035 (3)
O2'	0.130 (5)	0.074 (3)	0.155 (6)	0.029 (3)	0.050 (4)	−0.044 (4)
O3	0.0688 (12)	0.0459 (10)	0.0923 (15)	0.0231 (9)	0.0119 (11)	0.0035 (9)
O4	0.0524 (11)	0.0802 (14)	0.0776 (13)	0.0281 (10)	−0.0143 (9)	−0.0158 (11)
O5	0.0668 (12)	0.0628 (11)	0.0528 (10)	0.0211 (9)	0.0102 (9)	0.0050 (8)
N1	0.0435 (10)	0.0453 (10)	0.0535 (11)	0.0158 (8)	0.0049 (8)	0.0067 (8)
C1	0.0514 (15)	0.0519 (15)	0.0835 (19)	0.0159 (12)	−0.0037 (13)	−0.0059 (13)
C2	0.0452 (15)	0.078 (2)	0.133 (3)	0.0238 (15)	0.0088 (17)	0.000 (2)
C3	0.0696 (19)	0.083 (2)	0.075 (2)	0.0187 (16)	0.0019 (15)	0.0304 (17)
C4	0.093 (3)	0.132 (4)	0.094 (3)	0.005 (3)	−0.032 (2)	0.049 (3)
C5	0.0614 (15)	0.0504 (14)	0.0593 (15)	0.0212 (12)	0.0077 (12)	−0.0036 (11)
C6	0.091 (2)	0.0666 (17)	0.0684 (18)	0.0484 (17)	0.0055 (16)	0.0087 (14)
C7	0.0561 (16)	0.0599 (16)	0.089 (2)	0.0262 (13)	0.0178 (14)	0.0033 (14)
C8	0.081 (2)	0.079 (2)	0.177 (4)	0.048 (2)	0.043 (3)	0.011 (3)
C17	0.0716 (18)	0.0591 (15)	0.0453 (13)	0.0331 (14)	0.0073 (12)	−0.0049 (11)
C18	0.0551 (13)	0.0469 (12)	0.0403 (11)	0.0251 (10)	0.0063 (9)	0.0021 (9)
C19	0.0577 (16)	0.0710 (18)	0.0563 (15)	0.0214 (13)	−0.0099 (12)	−0.0125 (13)
C20	0.0465 (15)	0.086 (2)	0.085 (2)	0.0300 (15)	−0.0087 (14)	−0.0126 (17)
C21	0.0523 (15)	0.085 (2)	0.081 (2)	0.0394 (15)	0.0037 (14)	−0.0147 (16)
C22	0.0560 (15)	0.0758 (18)	0.0602 (15)	0.0365 (14)	−0.0032 (12)	−0.0226 (13)
C23	0.0435 (11)	0.0532 (13)	0.0450 (12)	0.0245 (10)	0.0016 (9)	−0.0021 (10)
N2	0.090 (4)	0.049 (2)	0.063 (5)	0.026 (2)	0.016 (4)	0.011 (3)
C9	0.115 (7)	0.104 (6)	0.181 (8)	0.036 (5)	−0.002 (6)	0.005 (6)
C10	0.182 (11)	0.226 (12)	0.227 (12)	0.106 (9)	−0.002 (8)	−0.015 (9)
C11	0.185 (8)	0.101 (6)	0.086 (5)	0.062 (6)	0.021 (5)	0.015 (4)
C12	0.196 (10)	0.140 (8)	0.097 (7)	0.073 (7)	0.028 (7)	0.000 (6)
C13	0.127 (6)	0.098 (5)	0.113 (6)	0.060 (5)	0.025 (5)	0.012 (4)
C14	0.192 (11)	0.198 (11)	0.214 (12)	0.092 (8)	0.007 (8)	0.006 (8)
C15	0.116 (5)	0.073 (4)	0.086 (4)	0.039 (4)	0.014 (4)	−0.003 (3)
C16	0.109 (6)	0.106 (6)	0.082 (5)	0.040 (5)	−0.002 (5)	−0.007 (4)

### Geometric parameters (Å, °)

**Table:**

S1—O3	1.432 (2)	C18—C19	1.387 (4)
S1—O5	1.4325 (19)	C18—C23	1.401 (3)
S1—O4	1.444 (2)	C19—C20	1.373 (4)
S1—S2	2.1075 (9)	C19—H19	0.9300
S2—C23	1.774 (2)	C20—C21	1.365 (4)
O1—C17	1.280 (5)	C20—H20	0.9300
O2—C17	1.188 (5)	C21—C22	1.370 (4)
O1'—C17	1.285 (6)	C21—H21	0.9300
O1'—H1'	0.8200	C22—C23	1.400 (3)
O2'—C17	1.217 (6)	C22—H22	0.9300
N1—C5	1.502 (3)	N2—C15	1.495 (9)
N1—C7	1.515 (3)	N2—C11	1.497 (9)
N1—C3	1.521 (4)	N2—C13	1.502 (9)
N1—C1	1.525 (3)	N2—C9	1.503 (10)
C1—C2	1.502 (5)	C9—C10	1.496 (9)
C1—H1A	0.9700	C9—H9A	0.9700
C1—H1B	0.9700	C9—H9B	0.9700
C2—H2A	0.9600	C10—H10A	0.9600
C2—H2B	0.9600	C10—H10B	0.9600
C2—H2C	0.9600	C10—H10C	0.9600
C3—C4	1.502 (5)	C11—C12	1.513 (9)
C3—H3A	0.9700	C11—H11A	0.9700
C3—H3B	0.9700	C11—H11B	0.9700
C4—H4A	0.9600	C12—H12A	0.9600
C4—H4B	0.9600	C12—H12B	0.9600
C4—H4C	0.9600	C12—H12C	0.9600
C5—C6	1.488 (4)	C13—C14	1.469 (9)
C5—H5A	0.9700	C13—H13A	0.9700
C5—H5B	0.9700	C13—H13B	0.9700
C6—H6A	0.9600	C14—H14A	0.9600
C6—H6B	0.9600	C14—H14B	0.9600
C6—H6C	0.9600	C14—H14C	0.9600
C7—C8	1.500 (4)	C15—C16	1.467 (8)
C7—H7A	0.9700	C15—H15A	0.9700
C7—H7B	0.9700	C15—H15B	0.9700
C8—H8A	0.9600	C16—H16A	0.9600
C8—H8B	0.9600	C16—H16B	0.9600
C8—H8C	0.9600	C16—H16C	0.9600
C17—C18	1.511 (3)		
			
O3—S1—O5	113.19 (13)	H8A—C8—H8C	109.5
O3—S1—O4	114.49 (13)	H8B—C8—H8C	109.5
O5—S1—O4	114.26 (13)	O2—C17—O2'	83.1 (5)
O3—S1—S2	107.77 (10)	O2—C17—O1	125.9 (3)
O5—S1—S2	107.23 (9)	O2'—C17—O1	73.1 (5)
O4—S1—S2	98.34 (9)	O2—C17—O1'	69.9 (4)
C23—S2—S1	105.73 (8)	O2'—C17—O1'	125.1 (4)
C17—O1'—H1'	120.0	O1—C17—O1'	85.3 (4)
C5—N1—C7	108.87 (19)	O2—C17—C18	120.9 (3)
C5—N1—C3	109.5 (2)	O2'—C17—C18	119.3 (4)
C7—N1—C3	111.1 (2)	O1—C17—C18	113.1 (3)
C5—N1—C1	111.6 (2)	O1'—C17—C18	115.6 (3)
C7—N1—C1	108.0 (2)	C19—C18—C23	118.6 (2)
C3—N1—C1	107.8 (2)	C19—C18—C17	118.6 (2)
C2—C1—N1	115.2 (3)	C23—C18—C17	122.8 (2)
C2—C1—H1A	108.5	C20—C19—C18	122.2 (3)
N1—C1—H1A	108.5	C20—C19—H19	118.9
C2—C1—H1B	108.5	C18—C19—H19	118.9
N1—C1—H1B	108.5	C21—C20—C19	119.2 (3)
H1A—C1—H1B	107.5	C21—C20—H20	120.4
C1—C2—H2A	109.5	C19—C20—H20	120.4
C1—C2—H2B	109.5	C20—C21—C22	120.3 (3)
H2A—C2—H2B	109.5	C20—C21—H21	119.8
C1—C2—H2C	109.5	C22—C21—H21	119.8
H2A—C2—H2C	109.5	C21—C22—C23	121.5 (2)
H2B—C2—H2C	109.5	C21—C22—H22	119.3
C4—C3—N1	115.2 (3)	C23—C22—H22	119.3
C4—C3—H3A	108.5	C22—C23—C18	118.2 (2)
N1—C3—H3A	108.5	C22—C23—S2	123.68 (19)
C4—C3—H3B	108.5	C18—C23—S2	118.15 (17)
N1—C3—H3B	108.5	C15—N2—C11	108.0 (8)
H3A—C3—H3B	107.5	C15—N2—C13	112.0 (9)
C3—C4—H4A	109.5	C11—N2—C13	115.3 (10)
C3—C4—H4B	109.5	C15—N2—C9	108.4 (10)
H4A—C4—H4B	109.5	C11—N2—C9	113.1 (10)
C3—C4—H4C	109.5	C13—N2—C9	99.7 (9)
H4A—C4—H4C	109.5	C10—C9—N2	115.5 (12)
H4B—C4—H4C	109.5	C10—C9—H9A	108.4
C6—C5—N1	115.4 (2)	N2—C9—H9A	108.4
C6—C5—H5A	108.4	C10—C9—H9B	108.4
N1—C5—H5A	108.4	N2—C9—H9B	108.4
C6—C5—H5B	108.4	H9A—C9—H9B	107.5
N1—C5—H5B	108.4	N2—C11—C12	117.8 (9)
H5A—C5—H5B	107.5	N2—C11—H11A	107.9
C5—C6—H6A	109.5	C12—C11—H11A	107.9
C5—C6—H6B	109.5	N2—C11—H11B	107.9
H6A—C6—H6B	109.5	C12—C11—H11B	107.9
C5—C6—H6C	109.5	H11A—C11—H11B	107.2
H6A—C6—H6C	109.5	C14—C13—N2	115.8 (11)
H6B—C6—H6C	109.5	C14—C13—H13A	108.3
C8—C7—N1	116.2 (3)	N2—C13—H13A	108.3
C8—C7—H7A	108.2	C14—C13—H13B	108.3
N1—C7—H7A	108.2	N2—C13—H13B	108.3
C8—C7—H7B	108.2	H13A—C13—H13B	107.4
N1—C7—H7B	108.2	C16—C15—N2	115.7 (8)
H7A—C7—H7B	107.4	C16—C15—H15A	108.4
C7—C8—H8A	109.5	N2—C15—H15A	108.4
C7—C8—H8B	109.5	C16—C15—H15B	108.4
H8A—C8—H8B	109.5	N2—C15—H15B	108.4
C7—C8—H8C	109.5	H15A—C15—H15B	107.4
			
O3—S1—S2—C23	67.06 (13)	C17—C18—C19—C20	−179.3 (3)
O5—S1—S2—C23	−55.11 (13)	C18—C19—C20—C21	0.3 (5)
O4—S1—S2—C23	−173.80 (13)	C19—C20—C21—C22	−2.0 (5)
C5—N1—C1—C2	55.0 (3)	C20—C21—C22—C23	2.4 (5)
C7—N1—C1—C2	174.6 (3)	C21—C22—C23—C18	−0.9 (4)
C3—N1—C1—C2	−65.3 (3)	C21—C22—C23—S2	178.8 (2)
C5—N1—C3—C4	62.1 (4)	C19—C18—C23—C22	−0.7 (4)
C7—N1—C3—C4	−58.2 (4)	C17—C18—C23—C22	179.7 (3)
C1—N1—C3—C4	−176.3 (4)	C19—C18—C23—S2	179.5 (2)
C7—N1—C5—C6	−59.0 (3)	C17—C18—C23—S2	−0.1 (3)
C3—N1—C5—C6	179.3 (3)	S1—S2—C23—C22	−3.3 (3)
C1—N1—C5—C6	60.1 (3)	S1—S2—C23—C18	176.46 (17)
C5—N1—C7—C8	−174.2 (3)	C15—N2—C9—C10	59.8 (16)
C3—N1—C7—C8	−53.5 (4)	C11—N2—C9—C10	−59.9 (17)
C1—N1—C7—C8	64.5 (4)	C13—N2—C9—C10	177.1 (14)
O2—C17—C18—C19	141.2 (5)	C15—N2—C11—C12	169.3 (12)
O2'—C17—C18—C19	41.1 (6)	C13—N2—C11—C12	43.1 (17)
O1—C17—C18—C19	−41.7 (4)	C9—N2—C11—C12	−70.8 (16)
O1'—C17—C18—C19	−137.8 (4)	C15—N2—C13—C14	−52.6 (15)
O2—C17—C18—C23	−39.2 (6)	C11—N2—C13—C14	71.5 (15)
O2'—C17—C18—C23	−139.3 (5)	C9—N2—C13—C14	−167.1 (13)
O1—C17—C18—C23	137.9 (4)	C11—N2—C15—C16	−172.6 (10)
O1'—C17—C18—C23	41.8 (5)	C13—N2—C15—C16	−44.6 (13)
C23—C18—C19—C20	1.1 (4)	C9—N2—C15—C16	64.5 (12)

### Hydrogen-bond geometry (Å, °)

**Table:**

*D*—H···*A*	*D*—H	H···*A*	*D*···*A*	*D*—H···*A*
O1'—H1'···O1^i^	0.82	1.62	2.436 (6)	175

Symmetry codes: (i) −*x*+1, −*y*+2, −*z*+2.
